# Management Challenges in a Child with Chronic Hyponatremia: Use of V2 Receptor Antagonist

**DOI:** 10.1155/2017/3757423

**Published:** 2017-01-09

**Authors:** Sowmya Krishnan, Swapna Deshpande, Ashwini Mallappa, Gunda Divya, Pascale Lane, Anu Vishwanath, Rene Y. McNall-Knapp

**Affiliations:** ^1^Department of Pediatrics, University of Oklahoma Health Sciences Center, Oklahoma City, OK, USA; ^2^Department of Psychiatry, University of Oklahoma Health Sciences Center, Oklahoma City, OK, USA; ^3^Department of Radiology, University of Oklahoma Health Sciences Center, Oklahoma City, OK, USA

## Abstract

Chronic hyponatremia is very rare in children and is often seen in the setting of congestive heart failure or liver failure in adults. Here, we report an 8-year-old child with hypothalamic glioma who presented with severe hyponatremia. Initial management consisted of fluid restriction. This was very difficult for the child to follow and the child developed bizarre drinking habits requiring intervention from child psychiatry. So therapy was initiated with low dose V2 receptor antagonist under close inpatient monitoring. While initial response was reassuring, her sodium levels tended to drift down with longer duration of treatment requiring us to increase the dose frequently. Her response to therapy and her stable clinical situation off therapy suggest that she may have reset osmostat.

## 1. Case

An 8-year-and-1-month old Caucasian female presented to her pediatrician with complaints of nocturnal enuresis and hyperactivity. Child was adopted at 2 years of age along with her younger sibling due to history of parental neglect by biological parents. Adoptive parents reported hyperactivity as a major concern. Physical examination revealed her weight to be 24.8 Kg (25th percentile for age) and height to be 128.7 cm (35th percentile for age). Vitals were stable and blood pressure at presentation was 113/75 mmHg. A comprehensive neurologic exam by her pediatrician revealed fine tremors in the upper extremity. Laboratory workup was initiated and a comprehensive metabolic panel revealed sodium (Na) of 124 mEq/L. She was referred to our medical center for comprehensive evaluation. Initial exam revealed an otherwise healthy child who was alert and oriented in time, space, and person. Vitals including blood pressure were normal for age. Admission chemistry and other laboratory values are shown in Table 1.

**Figure 1:**
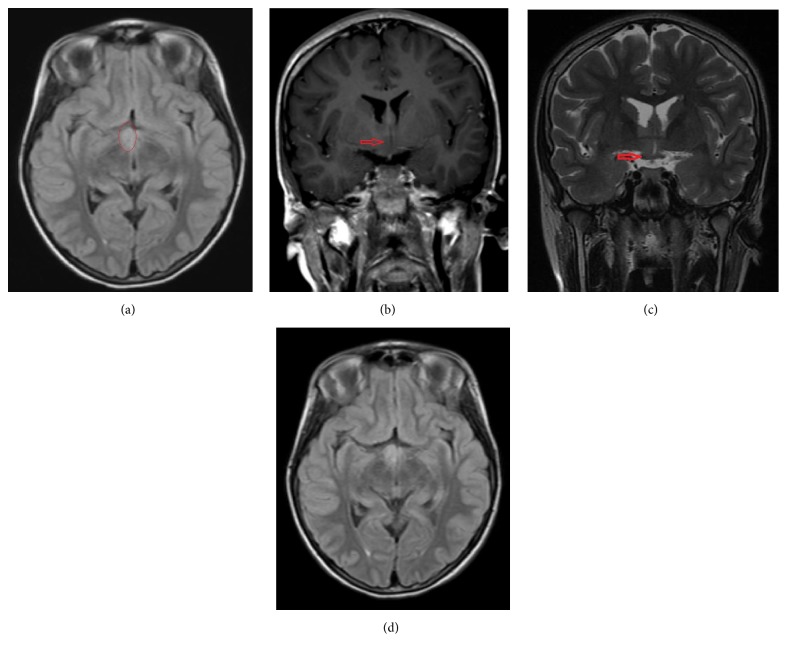
MRI of the brain. (a) Axial FLAIR image demonstrates asymmetric enlargement with abnormal FLAIR hyperintense signal within the right hypothalamus (red circle). (b) Coronal postcontrast T1 weighted image demonstrates no abnormal enhancement within the enlarged right hypothalamus (red arrow). (c) Coronal T2 weighted image demonstrates the mass abutting the lateral margin of the third ventricle and the superior margin of the optic chiasm (red arrow). (d) Follow-up axial FLAIR image, approximately 3 years later, demonstrates no significant change in size and abnormal hyperintense signal within the lesion.

While the causes of hyponatremia can be extensive [1, 2], her presentation was consistent with euvolemic hyponatremia suggestive of Syndrome of Inappropriate Antidiuretic Hormone (SIADH). Evaluation by us confirmed our impression of SIADH with serum Na of 123 mEq/L, serum osmolality of 264 mOsm/Kg (ref range 280–300), and urine osmolality of 614 mOsm/Kg (300–900). Adrenal function as assessed by early morning cortisol of 8.6 mcg/dL and thyroid function tests were normal. Magnetic resonance imaging of the brain demonstrated asymmetric T2/FLAIR hyperintense, nonenhancing enlargement of the right hypothalamus, concerning for a hypothalamic glioma (Figure 1). Other workups including ultrasound of abdomen, pelvis, chest X-ray, and a PPD (purified protein derivative) skin test to rule out tuberculosis were essentially negative.

Initial management consisted of fluid restriction, furosemide (at a dose of 2 mg/Kg/day divided into three doses), and salt and potassium chloride supplementation. Though this resulted in her Na levels gradually improving to a maximum value of 130 mEq/L, the fluid restriction regimen was very difficult to follow and family expressed ongoing frustration. Given limitations for treatment options in treating children with SIADH and with Na levels trending down (range: 121–124 mEq/L), we initiated Arginine Vasopressin (AVP) V2 receptor antagonist therapy with oral tolvaptan. Current literature is lacking data on use of these agents in children. So tolvaptan therapy was started at a dose of 7.5 mg under close supervision in the inpatient setting. The dose initiated was half of the lowest approved dose in adults which is 15 mg [3]. Initial response to therapy was dramatic in terms of correction of hyponatremia, with Na improving to low 130 mEq/L range.

With initiation of tolvaptan therapy, her Na levels improved allowing us to loosen our recommendation on water intake to a maximum of 2.5 L/day. Liberalization of fluid intake with this regimen seemed to help the family initially. Gradually though, Na levels trended down because her water intake was much more than her urine output with the therapy (Table 2). Child psychology and later child psychiatry were involved to help family and child be compliant with the fluid restriction. Dose of tolvaptan had to be increased gradually to a maximum of 30 mg daily. In an effort to keep the Na levels up, water restriction to 2.5 L/day was encouraged. But even this degree of fluid restriction proved to be difficult as she developed compulsive drinking behaviors. She would sneak water from bottles in trash cans, bathtub, faucet, and even toilets.

With ongoing management issues due to behavior problems and recent black box warning issued by the Food and Drug Administration (FDA) for liver failure in patients on long-term therapy [3], decision was made to wean tolvaptan. She was weaned off V2 antagonist therapy over a period of 6 months. While her persistent hyponatremia was concerning, the limited benefits we were observing with treatment and the potential side effects led us to make this decision. Following this, patient was briefly on oral furosemide, which was eventually discontinued as long-term furosemide is known to exacerbate chronic hyponatremia [4].

She was also noted to develop hypertension with blood pressures ranging around 140/106 mmHg requiring input from pediatric nephrology during the course of her treatment. She was initially started on amlodipine 2.5 mg twice a day but needed the addition of enalapril maleate in 4 months due to poor control of her blood pressure. She is currently on amlodipine 2.5 mg twice a day and enalapril 10 mg twice a day. Secondary causes of hypertension were ruled out by pediatric nephrology team. While hypertension was noticed after initiation of therapy, this is not a reported side effect of V2 receptor antagonist therapy. Her hypertension continued after she was weaned of tolvaptan therapy. It is unlikely that the hypertension was caused by volume overload as her weight continued to be stable. Etiology of hypertension is unclear as preliminary workups including renal ultrasound and renal Doppler were all within normal limits.

A complete neuropsychological assessment revealed IQ to be low normal. Patient had reading, attention, and auditory processing problems making school more challenging for her. She had no difficulties in doing grade level mathematics and spelling. These contributed to ongoing difficulties at school. Due to the well-known determinant effect of hyponatremia on bone health [5], DXA scan was done which revealed* Z*-score of −1.8 at AP spine and −1.1 at total body less head region. Since she never suffered from any pathological fracture, education was provided to optimize calcium and vitamin D intake. 25-Hydroxyvitamin D levels checked periodically were always in the sufficient range (>30 ng/mL).

Currently, she is off all pharmacological therapy for her hyponatremia and her sodium levels range within 113–118 mEq/L. Fluid restriction is encouraged but not very strictly enforced due to fear of her developing unhealthy drinking habits. She is performing at age appropriate level in school. Regular monitoring for endocrinopathies by stimulation testing has so far been normal.

## 2. Discussion

Our patient presented with classical features of SIADH secondary to a low grade hypothalamic glioma. Her subsequent clinical course and poor response to tolvaptan suggest that she may have reset osmostat. Increase in AVP levels with initiation of treatment suggested reset osmostat driving the increased thirst and AVP levels. Fractional excretion of uric acid was 11% favoring reset osmostat as the diagnosis, although this was calculated while she was on tolvaptan therapy. Reset osmostat has been described primarily in elderly people with underlying medical condition like paraplegia and spinal trauma [6]. Additionally, hyponatremia in these cases tends to be mild. While there is a case report of an 8-month-old child with reset osmostat, that child had additional morphological abnormalities like cleft palate [7, 8]. To our knowledge, this is the only case report of a child with severe hyponatremia caused by a reset osmostat due to low grade hypothalamic glioma. Elevated AVP levels are often seen in the setting of hyponatremia even if the cause is not SIADH [9]. While there are multiple pitfalls in the evaluation of a patient with hyponatremia [10], we were able to avoid these and longitudinal follow-up over a long period of time helped us establish the diagnosis.

The bizarre drinking behavior exhibited by this patient could also be because of pathological thirst control secondary to the glioma which is then causing the persistent hyponatremia. However the rise in AVP levels that we saw with treatment with tolvaptan suggests that she has a reset osmostat. However, a coexisting pathological thirst control cannot be completely ruled out. Pathological thirst with severe hyponatremia has been previously described in a 26-year old as sequelae of viral encephalitis [11]. In the setting of hyponatremia and compulsive drinking, psychogenic polydipsia can be considered in the differential diagnosis after ruling out other medical causes for this. Literature is scarce on this disorder in children, but, in adults, it is often seen in schizophrenia and other psychiatric disorders [12].

The patient's hypothalamic glioma extended along the anterolateral margin of the third ventricle, which contains the paraventricular nuclei responsible for secreting AVP** (**Figure 1**)**. Additional differential considerations for her hypothalamic lesion include a hypothalamic hamartoma or other infectious/inflammatory lesions. Hypothalamic hamartomas, although likely in this patient, typically present with gelastic seizures and precocious puberty and tend to be isointense to gray matter [13]. Other infectious/inflammatory etiologies would be considered at initial presentation, but the absence of additional abnormalities on the brain MRI and lack of enhancement and stability of the lesion made this a less likely possibility. Therefore, a hypothalamic glioma was the favored diagnosis. Tumor board discussion was held to discuss potential therapy for presumed hypothalamic low grade glioma. The tumor board was attended by a neuroradiologist, radiation oncologists, pediatric oncologists, a pediatric neurosurgeon, and her endocrinologist. Given the proximity of the lesion to the optic chiasm and lack of progression, a biopsy or resection was deferred to spare the risk of vision loss. Follow-up brain MRIs over a four-year time course demonstrated very minimal, if any, interval growth over serial studies** (**Figure 1**)**. No additional lesions were identified. The lack of interval growth of the lesion also favored against pursuing aggressive therapy such as radiation, which carried its own complications of radiation necrosis, secondary tumors, and moyamoya disease.

V2 receptor antagonist was well tolerated in our patient and was effective in raising sodium levels in the short term. But long-term management may be complicated by liver problems including liver failure. Restricting water intake continues to be the cornerstone in the management of SIADH, though this is very difficult to implement in children. Behavioral interventions of implementation of reinforcement schedule that rewards compliance and cognitive behavioral therapy with higher functioning individuals have been shown to help with fluid restriction [12].

## Figures and Tables

**Table 1:** Serum and urine chemistry at initial presentation.

Lab parameter	Value
Serum sodium (mEq/L) (134–145)	123
Serum potassium (mEq/L)	5.2
Serum chloride (mEq/L)	90
Serum bicarbonate	23
Anion gap	8
Blood urea nitrogen	6
Serum creatinine	0.4
Serum glucose (mg/dL)	105
Serum calcium (mg/dL)	9.2
Uric acid (mg/dL) (2.6–6.0)	1.6
Plasma renin activity (ng/mL/hr)	<10
Aldosterone (ng/dL)	11
Serum osmolality (mOsm/Kg) (280–300)	264
Urine osmolality (mOsm/Kg) (300–900)	614
Urine Na (mEq/L)	122
Arginine vasopressin (pg/mL) (<7.0)	7.8

**Table 2:** Clinical course with initiation of tolvaptan therapy.

Timeline	Weight (Kg)	Sodium (mEq/L)	Tolvaptan dose	Serum osmolality (mOsm/Kg)	Urine osmolality (mOsm/Kg)	AVP (pg/mL)
Prior to initiation of tolvaptan therapy	24.7	121	None	263	428	11.1
Day 2 of initiation of therapy	24.8	127	7.5 mg		26	43.4
6 months after initiation	25.6	123	Increased to 15 mg	250	55	3.0
7 months after initiation	25.9	123	Increased to 22.5 mg			
17 months after initiation	26	119	Increased to 30 mg	253		19.5
24 months after initiation	28.6	124	Tolvaptan gradually stopped			
1 year after stopping tolvaptan therapy	31.4	118-119	None	235	242	2.2
