# Corrigendum to “Management Challenges in a Child with Chronic Hyponatremia: Use of V2 Receptor Antagonist”

**DOI:** 10.1155/2017/8132129

**Published:** 2017-07-02

**Authors:** Sowmya Krishnan, Swapna Deshpande, Ashwini Mallappa, Divya Gunda, Pascale Lane, Anu Vishwanath, Rene Y. McNall-Knapp

**Affiliations:** ^1^Department of Pediatrics, University of Oklahoma Health Sciences Center, Oklahoma City, OK, USA; ^2^Department of Psychiatry, University of Oklahoma Health Sciences Center, Oklahoma City, OK, USA; ^3^Department of Radiology, University of Oklahoma Health Sciences Center, Oklahoma City, OK, USA

In the article titled “Management Challenges in a Child with Chronic Hyponatremia: Use of V2 Receptor Antagonist” [1], the name of the fourth author was reversed. The author's name should have been written as Divya Gunda. The revised authors' list is shown above.
